# Strengthening the ICUs' human resource‐related responses to Covid‐19: A rapid review of the experience during the first year of public health emergency

**DOI:** 10.1002/hpm.3569

**Published:** 2022-09-27

**Authors:** Aizhan Tursunbayeva, Stefano Di Lauro

**Affiliations:** ^1^ University of Naples Parthenope Naples Italy; ^2^ University of Sannio Benevento Italy

**Keywords:** Covid‐19, health, management, planning, workforce

## Abstract

By drawing on macro‐categories of key human resource (HR) management interventions recommended by the Organization for Economic Co‐operation and Development (OECD) during the Covid‐19 pandemic, this study aimed to explore whether and how Intensive Care Units (ICU) have strengthened their HRs during the first year of Covid‐19 emergency. A rapid review was conducted to provide a quick synthesis of the literature in English identified in the Web of Science Core Collection (WoS), PubMed, and Scopus databases. A total of 68 articles qualified for the final analysis. The findings illustrated that health organisations were often guided by staffing ratios to estimate capacity to care, aimed to modify the scope of practice of providers, redeployed both internal and external staff to ICUs, created and adapted the Covid‐19‐specific staffing models, and implemented technological innovations to provide services to the unprecedented number of patients while protecting the physical and mental health of their staff. The insights of this research should be helpful for health leaders, HR Managers, and policymakers who have faced unprecedented challenges and tough decisions during this emergency. The findings could also inform beyond‐Covid‐19 ICU policies and guide future research.

## Highlights

- This study presents state‐of‐the‐art strategies to mobilise Intensive Care Units' (ICU) staff, revisit scope of practice (SOP), and protect the physical and mental health of employees.
- Existing research on ICUs' human resource (HR)‐related responses is predominantly monodisciplinary, comes primarily from medical journals, and mostly describes the experiences of ICUs within individual health organisations from high‐income countries.
- The literature examined was mostly silent about broader HR management‐related practices, and the relevant important role HR practitioners play.
- The insights of this research should be helpful for multidisciplinary actors, inform beyond‐Covid‐19 ICU policies and guide future research.

## 1 BACKGROUND

An ongoing public health emergency of international concern was triggered on 30 January 2020 by the Covid‐19 virus. The epidemiology of the pandemic has contributed to an unprecedented increase in the volume of demand on the health systems that is likely to continue for the near future.^1^ Some of the Covid‐19 patients are asymptomatic, ‘mild’ Covid‐19 cases can recover at home, while more complex cases may require hospital care or, in critical circumstances, ICU admission.^2^ The rate of ICU admissions among patients with Covid‐19 has been varying between 9% and 26% with a median length of stay of nine days,^3^ presenting a unique challenge to hospitals worldwide running short of their ICU beds.^4^, ^5^

A range of policy options has been explored to rapidly increase the number of ICU beds internationally. For example, after years of healthcare‐related funding cuts, the government of Italy has unlocked resources to double the number of its ICU beds (from 5,300 to 11,060)^6^ to meet the growing demand. However, one of the biggest challenges the governments and health systems have faced in expanding existing or creating new ICUs was staffing them with trained medical and nursing staff ready to take care of Covid‐19 patients. ‘Care is about more than a room with a hospital bed. It's about medical professionals taking care of patients’,^7^ and health staff, unlike ICU beds or ventilators, cannot be produced suddenly or work at maximum occupancy for long periods.^8^ Indeed, a recent survey of ICU directors found that ICU bed capacity could be increased by as much as 191%, invasive ventilator numbers by only 120%, but the surge would require 325% of additional senior doctors and 365% of registered ICU nurses.^9^

Moreover, existing ICU health staff working on the frontlines themselves fall sick or must quarantine themselves after being exposed to infected people, which further have reduced the total number of staff available.^10^ Thus, comprehensive ICU staff management and planning have emerged as a critical factor in the management of the outbreak. Nevertheless, despite a growing number of studies on Covid‐19, very little is still known about this specific topic.

This paper aim to address this knowledge gap. Specifically, this study located, analysed, and synthesised existing international evidence on the strategies adopted or proposed for adoption to strengthen the HR of ICUs during the first year of the Covid‐19 emergency, which have not been addressed elsewhere to the best of our knowledge. Finally, this paper also identifies and discusses key themes and gaps in the literature.

## 2 METHODS

Given the still ongoing and changing nature of the Covid‐19 pandemic, health managers and policymakers require a review and synthesis of the evidence‐based literature to inform their practice. The WHO advocates rapid reviews in these circumstances,^11^ with simplified components of systematic reviews. Rapid reviews are generally recommended to study changing phenomena and provide state‐of‐the‐art answers to the question of interest, as well as to identify the gaps in the literature. Thus, it was considered a valid method to explore the approaches adopted or proposed for adoption to strengthen the HR of ICUs during the first year of the Covid‐19 emergency. An eight‐stage rapid review methodology, described below, was used to standardise and clarify the procedures.^12^

Stage 1. Review initiation

This review was conducted by two researchers experienced in interdisciplinary systematic and scoping review methodologies, who also have practical and research experience in the fields of HR and/or health HR management (HRM).

Stage 2. Review question

In general, literature reviews with more focussed questions require fewer resources and a shorter time for completion. Since rapid reviews aim to summarise a certain amount of literature on a given topic relatively quickly, we focussed on a specific research question: ‘Approaches adopted to strengthen the HR in ICUs in response to Covid‐19’.

Stage 3. Identification of relevant studies

A search strategy was developed and tested iteratively to locate relevant literature in the WoS, Scopus, and PubMed interdisciplinary literature databases. The former two were reported to be well‐suited to synthesise evidence in the form of literature reviews,^13^ while the latter one is a well‐known resource for literature in health and medicine.

**FIGURE 1:**
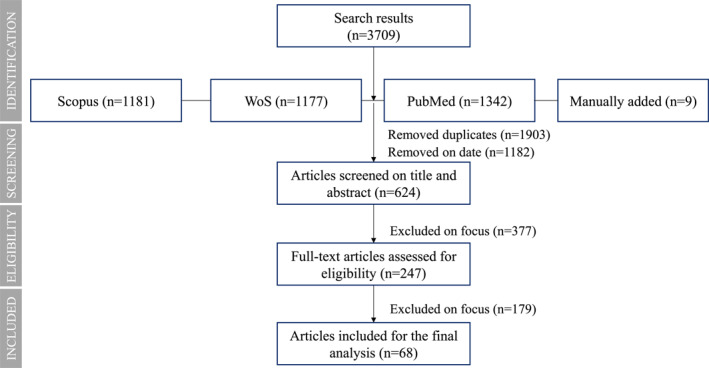
PRISMA diagram

These databases were searched in May 2022 with a structured search query including ‘Intensive care unit’ OR ICU AND Covid keywords. Generated returns (specifically those published between 2020 and 2021) were extracted in EPPI‐Reviewer software, which was later used for screening. PubMed was searched via EPPI directly. Articles not in English were not included in the analysis. Initially, the articles were screened on the publishing date. Here only articles published between 1 January 2020 and 30 January 2021 were included in further screening first on the titles and abstracts and later on their full text (i.e., to determine their relevance to the review's purpose and research question). Articles deemed outside the scope of the review (i.e., ones not related to the aspects of ICU HR) were excluded. The process of study selection was charted using a PRISMA diagram (see Figure 1).

Stage 4. Description of study characteristics

For the included on the full‐text articles publication characteristics including journal name, publication year, setting (health organization or the country in which the study was conducted), journal discipline, article type, and study design were extracted to the pre‐defined spreadsheet manually.

Stage 5. Quality assessment

A quality assessment of the literature was not performed as the intent of this study was to inclusively emerge patterns rather than to assess the available evidence.

Stage 6. Data extraction

The main data extraction included summarising key characteristics of eligible publications into an Excel spreadsheet. One author extracted the data from all eligible studies. Another author double‐checked extracted information to ensure no data was missing.

Stage 7. Synthesis of findings

Predefined categories guided the data extraction stage. Thus, the disciplinary affiliation of journals was assessed with reference to their classification in Scimago,^14^ while the countries of research were classified according to the World Bank's Country and Lending Groups.^15^ The study findings were grouped into macro‐categories of key HR management areas as recommended by the OECD, such as mobilising health professionals, adapting the roles and responsibilities of providers, and protecting the health of health workers from Covid‐19.^16^

Stage 8. Using the review

The use of a PRISMA diagram and structured process aimed to ensure that the review was transparent, accountable,^12^ and replicable. The review results that are summarised and discussed below should be of interest to governments and health leaders, policymakers, and interdisciplinary scholars.

## 3 RESULTS

### 3.1 Publication characteristics

Searching Scopus, WoS, and PubMed using a structured query yielded 3,700 results in English. Nine additional publications that the authors were familiar with based on the background readings were added to these results.

After removing duplicates (*n* = 1,903), and articles not published between 1 January 2020 and 30 January 2021, 624 returns remained for screening on titles and abstracts. Of these, 247 full‐text articles were assessed for eligibility. After excluding articles not related to the ICU HR management during Covid‐19, 68 articles qualified for the final analysis.

A total of 84% of qualifying publications was published in academic journals listed in Scimago. The analysis of the journal disciplines revealed that 77% of these articles were published in monodisciplinary journals (84% in medical and 16% in nursing), while 23% appeared in interdisciplinary journals with a focus on medicine and nursing; medicine, pharmacology, toxicology, and pharmaceutics; medicine and neuroscience; agricultural and biological sciences, and biochemistry, genetics and molecular biology; and health professions, medicine and nursing.

Of the eligible publications, 18% were quantitative, qualitative or mixed‐method studies, and 7% were literature reviews. The remaining publications were discussion papers or case reports revealing experiences related to reorganization, the creation of new ICUs, or health professionals' experience with working in ICUs during Covid‐19.

The country of research was specified in 76% of studies (see Table 1). Most of them came from the US (26%), and Europe and Central Asia (25%).

**TABLE 1:** Region, country, and type of economy

Region and type of economy	Country	%	References
North America			
High‐income economy	US	25.4	^17^, ^18^, ^19^, ^20^, ^21^, ^22^, ^23^, ^24^, ^25^, ^26^, ^27^, ^28^, ^29^, ^30^, ^31^, ^32^, ^33^, ^34^
Europe and Central Asia			
High‐income economy	Italy	7.0	^35^, ^36^, ^37^, ^38^, ^39^
	UK and Ireland	7.0	^40^, ^41^, ^42^, ^43^, ^44^
	France	4.2	^45^, ^46^, ^47^
	Spain	1.4	^48^
	Belgium	1.4	^49^
Upper‐middle‐income economy	Turkey	1.4	^50^
N/A	Generic	1.4	^51^
East Asia and Pacific			
High‐income economy	Australia	5.6	^51^, ^52^, ^53^
	Singapore	5.6	^54^, ^55^, ^56^, ^57^
	Japan	1.4	^58^
	New Zealand	1.4	^51^
Upper‐middle‐income economy	China	4.2	^59^, ^60^, ^61^
South Asia			
Lower‐middle‐income economy	India	2.8	^62^, ^63^
Lathin America and the Caribbean			
Upper‐middle‐income economy	Brazil	1.4	^64^
Middle East and North Africa			
High‐income economy	Bahrain	1.4	^65^
	Libya	1.4	^66^
Upper‐middle‐income economy	Lebanon	1.4	^67^
N/A	N/A	22.9	^68^, ^69^, ^70^, ^71^, ^72^, ^73^, ^74^, ^75^, ^76^, ^77^, ^78^, ^79^, ^80^, ^81^, ^82^, ^83^

Another 51% of the articles specified the name and/or the type of health organisations they were focussed on.^17^, ^18^, ^19^, ^20^, ^21^, ^23^, ^26^, ^27^, ^28^, ^32^, ^33^, ^34^, ^35^, ^36^, ^37^, ^38^, ^39^, ^40^, ^43^, ^45^, ^46^, ^47^, ^48^, ^49^, ^53^, ^54^, ^55^, ^56^, ^57^, ^59^, ^60^, ^61^, ^62^, ^63^, ^64^, ^66^ All of the remaining studies did not specify a health organization of focus or concentrated on groups of respondents within health organisations.

### 3.2 Mobilising health professionals

Various qualifying studies reported on the ICU staffing ratios used during the Covid‐19 pandemic to estimate the number of personnel to mobilise (see Table 2). Many of them reported on the nurse‐to‐patient ratio, which was 1 nurse per 2 patients on average, and on the ICU physicians/intensivists‐to‐patient ratio, which varied, with 1 specialist for around 4–11 patients. Some also mentioned staffing ratios for respiratory therapists (e.g., 1 per 4–6 patients), Advanced Practice Providers (APP) (e.g., 1 per 5–7 patients), and pharmacists (e.g., 1 per 8–30 patients).

**TABLE 2:** ICU staffing ratios as reported in the included studies

Region, country (type of economy)	Health Organization	Staffing ratio (Nurse: Patient)	Staffing ratio (Physician/Intensivist: Patient)	Staffing ratio (Other Staff: Patient)	Reference
North America					
US (High‐income)	Recommendations of Healthforce Centre at UCSF	1:1–3^a^	Physician/intensivist: 1:7–10^a^	APP: 1:5–7^a^	^24^
				Respiratory therapist: 1:4–6^a^	
				Certified nursing assistant: 1:7–17^a^	
US (High‐income)	State‐level estimates	1:1–3^a^	Intensivist: 1:7–10^a^	Respiratory therapist: 1:4–6^a^	^29^
				Pharmacist: 1:8–30^a^	
US (High‐income)	Houston Methodist Hospital	1:2	Intensivist: 1:8–18^a^	N/A	^28^
US (High‐income)	New York‐Presbyterian Columbia University Irving Medical Centre	1:2–3^a^ (at peak with support of non‐critical care nurse)	N/A	N/A	^32^
US (High‐income)	State‐Level Estimates	N/A	Physician: 1:8–11 (some states 1:15)	N/A	^25^
US (High‐income)	Einstein Medical Centre	1:2	N/A	Respiratory therapist: 1:8	^20^
Europe and Central Asia					
Belgium (High‐income)	Three Hospitals In French‐Speaking Belgium	Legal ratio: 1:3 recommended ratio: 1:1	N/A	N/A	^49^
France (High‐income)	Gustave Roussy Cancer Campus	1:2 (with one extra nurse occasionally)	N/A	Caregiver to patient: 1:4	^45^
Italy (High‐income)	Large, Multidisciplinary, Academic Hospital	1:2^b^	1:5–6^b^	N/A	^36^
Italy (High‐income)	Maggiore della Carità University Hospital	1:2	Physician: 1:4	N/A	^38^
Italy (High‐income)	1000‐Bed Academic Hospital Located In North‐East Italy	1:3 (with three nursing assistants per shift)	N/A	N/A	^39^
UK^c^ (High‐income)	NHS England in Collaboration with Key Nursing Organisations	1:2 (with the support of a non‐critical care nurse)	N/A	N/A	^43^
South Asia					
India (Lower‐middle‐income)	Temporary structure	1:1–1.5 per ventilated patient	N/A	N/A	^62^
East Asia and Pacific					
China (Upper‐middle‐income)	Tongji Hospital (Guanggu Campus)	Government bed‐to‐care ratio is 1:6 (i.e., 180 nurses provide nursing for 30 patients)^b^	N/A	N/A	^60^
Singapore (High‐income)	Largest Academic Tertiary Medical Centre	Circa 1.5:1 (with the support of a non‐critical care nurse)	N/A	N/A	^55^
Singapore (High‐income)	The Singapore General Hospital	1:1	N/A	N/A	^57^
Middle East and North Africa					
Libya (Upper‐middle‐income)	16 leading Hospitals	62.5% = 1:4	N/A	N/A	^66^
		37.5% = 1:2			
Others					
N/A	N/A	1:1‐6^a^	N/A	N/A	^69^
Suggestion		2:1 when PPE is worn			
N/A	N/A	Staff to patient ratio: 1:2			^74^
Theoretical scenario					

^a^ The numbers can vary depending on the surge (min/max).

^b^ 4 shifts a day system, with teams handing over every 6 h.

^c^ The guidance will only come into force when an ICU exceeds capacity and is forced to open additional beds, otherwise the established 1:1 nurse‐to‐patient ratio will apply.

Many studies also testified that staff ratios were revisited during the pandemic. For example, some amended the nurse per patient ratios to address the surge, though noting that in this case, ICU nurses needed to be supported by non‐critical care nurses (e.g., for preparing drugs and equipment).^32^ For example, the Chair of the British Association of Critical Care Nurses stated, ‘We know that the evidence supports 1:1 ICU nurse ratios, we know that that means that we can deliver better care and we know that protects our staff…. We've got two options: we either accept that we are going to have to change our staffing model, or we turn patients away at the door…. And we absolutely cannot do that, so clearly we have to look at our nurse staffing model’.^43^ In the meantime, it was recommended in Belgium to increase the staff‐per‐patient ratio from the one recommended by the Belgian legislation (i.e., 1:3) to a 1:1 ratio to meet the demand of the Covid‐19 patients as these patients apparently required more time for monitoring, mobilisation, and hygiene care,^49^ while some proposed a more agile approach: revisiting the ratios frequently during the pandemic.^55^

The analysis also revealed additional approaches that health organisations adopted in their workforce planning sometimes in addition to or in combination with staffing ratios (e.g.,^28^, ^32^, ^45^, ^62^). For example, they considered the number of health staff per ICU bed^33^, ^62^ or for ICUs in general^32^, ^34^ per shift,^82^ used team‐based workforce models with multi‐disciplinary teams,^17^, ^69^ tiered staffing models (i.e., with ICU nurses guiding and supporting non‐ICU nurses),^19^ or grouped staff according to their experience or tasks^52^, ^77^ such as intubation, proning and turning, transfer, medication, hygiene, or ‘expert senior’ support teams.^52^ Finally, staffing models tied to epidemiological considerations (e.g.,^74^), demand for health services (e.g.,^20^, ^29^, ^34^, ^61^), or with the high standards of critical care and infection control^82^ were also suggested during the pandemic. Interestingly, it was reported that during Covid‐19, there was a reduction in the use of unscheduled benefit time and absences during the height of the Covid‐19 surge in some ICU units.^19^

The majority of studies analysed proved that throughout the pandemic, ICU workforce capabilities have been overwhelmed, making it necessary to draw the workforce (with and without critical care experience) from various departments and divisions that suspended their elective procedures or non‐essential services. For example, the All India Institute of Medical Sciences trained and deployed 25% of their resident doctors from each department to take care of critical Covid‐19 patients.^63^ The analysis helped to reveal specific categories of physicians, nurses, and other health personnel who were mobilised to support the ICUs (see Table 3).

**TABLE 3:** Types of physicians, nurses, and other staff redeployed to ICUs during Covid‐19 pandemic

Specialists	%	References
Physicians		
Intensivists	9	^32^, ^35^, ^45^, ^55^, ^56^, ^62^, ^66^, ^73^
Anesthesiologists	13	^20^, ^26^, ^27^, ^30^, ^33^, ^37^, ^38^, ^40^, ^41^, ^56^, ^64^, ^66^
Surgeons	8	^20^, ^21^, ^28^, ^30^, ^40^, ^45^, ^56^
Otolaryngologists	3	^21^, ^22^, ^26^
Ophthalmologists	2	^21^, ^63^
Gastroenterologists	1	^37^
Radiation oncologists	1	^21^
Physiatrists	1	^21^
Urologists	1	^21^
Dermatologists	1	^21^
Orthopedists	1	^21^
Pulmonary critical care doctors	2	^20^, ^34^
General practitioners	2	^56^, ^66^
Physicians/Doctors (generic)	3	^20^, ^34^, ^56^
Nurses (from)		
Critical care/Emergency rooms	12%	^9^, ^20^, ^21^, ^23^, ^32^, ^35^, ^36^, ^45^, ^55^, ^66^, ^77^
Surgical, post‐anaesthesia teams/rooms	8%	^17^, ^30^, ^33^, ^35^, ^38^, ^40^, ^69^
Subspeciality centres (e.g., infection control)	3%	^36^, ^56^, ^66^
Neurology and neuroscience departments	2%	^19^, ^60^
Oncology/haematology wards	1%	^45^
Nurse assistants	1%	^34^
Other staff		
Allied health professionals^a^	9%	^21^, ^27^, ^33^, ^34^, ^52^, ^56^, ^64^, ^66^
Palliative carers	6%	^21^, ^28^, ^30^, ^32^, ^33^
Pharmacists	3%	^21^, ^33^, ^48^
Paramedical staff	1%	^62^
Medical educators	1%	^52^
Social workers	1%	^33^
Students	1%	^18^

^a^ Respiratory therapists, physiotherapists, dieticians, and X‐ray technicians.

Only a few studies^26^, ^30^, ^45^, ^69^ mentioned the approach to the redeployment of these staff members (e.g., voluntarily). Many health organisations also mobilised external staff resources including the army, navy, or air force personnel (e.g.,^26^, ^62^); internationally educated healthcare workers (e.g.,^78^); students (e.g.,^18^, ^73^, ^83^); residents (e.g.,^20^); out‐of‐state staff^19^; and retirees (e.g.,^19^, ^55^, ^57^). Staff was also seconded from private hospitals (e.g.,^55^, ^57^, ^64^, ^83^), while the most tech‐savvy health organisations introduced virtual ICUs and embraced virtual staffing (e.g.,^31^), where staff who might be at high risk or had tested positive for Covid‐19 could provide care remotely without entering an ICU or even a hospital.^18^, ^28^ For example, in one of the US health care settings, 300 clinicians got access to technology that enabled them to care for patients remotely, as well as allowed 20 ICU nurses to work from home.^23^

### 3.3 Adapting the roles and responsibilities of providers

Some qualifying studies mentioned that healthcare workers and trainees were (or could be) temporarily redeployed to ICUs (often on very short notice^17^) even if the ICU was outside their normal SOP (e.g.,^22^, ^30^, ^32^, ^36^, ^77^, ^78^) or highlighted the importance of creating policies and guidelines for those who were working outside their normal minimum workforce standards guidelines.^52^ For instance, diverse US states (e.g., Pennsylvania, Tennessee, Wisconsin, Arizona, Michigan, and California) relaxed supervision and delegation requirements for a wide spectrum of health professionals after the invitation to do so by the US Secretary of Health and Human Services for all states, thereby allowing doctors to spend more of their time on the most complex cases (e.g.,^72^). These modifications were projected to be temporary, although the exact time frame for these adjustments was not firmly determined.

It was observed that the success of temporarily redeploying staff to ICUs in roles outside their SOP is not yet supported by high‐quality evidence,^78^ and that mobilised staff should not be expected to work outside their professional SOP independently unless they have been assessed as competent.^69^ In general, several authors recommended providing training for redeployed staff, especially to those who do not usually practice within hospital or ICU settings,^17^, ^19^, ^21^, ^25^, ^35^, ^38^, ^40^, ^42^, ^44^, ^46^, ^53^, ^54^, ^55^, ^56^, ^57^, ^59^, ^61^, ^62^, ^63^, ^64^, ^66^, ^69^, ^73^, ^77^, ^78^, ^82^ pairing them with core ICU staff (e.g.,^17^, ^19^, ^30^), or making the core staff act as ‘coaches’^19^, ^55^ to help with the acclimatization of redeployed staff. Though the articles did not specify whether or how such practices were formalised. Defining the new team member's role was also recommended at the outset,^32^ and the use of existing management tools was noted as helpful for analysing staff activities.^59^

Anecdotally, some articles mentioned that staff redeployed to ICUs during Covid‐19 (e.g., nurses) believed that they gained valuable knowledge and experience that could be beneficial for their future careers.^17^, ^39^ Meanwhile, there were also discussions around the ethical aspects of the providers' roles and responsibilities (i.e., fundamental Hippocratic ethical principles of beneficence, non‐maleficence, justice and respect for autonomy) who sometimes were unable to provide ICU care to all patients due to the high surge and/or unavailability of sufficient ICU beds.^81^

### 3.4 Protecting the health of health workers

High workloads and anxiety over Covid‐19 transmission can result in significant physical and mental fatigue and have behavioural and interpersonal effects on frontline staff including avoiding responsibility, or social interaction, respectively.^50^ The findings that emerged regarding the physical and mental protection of ICU HR are summarised in Table 4 and discussed in detail below.

**TABLE 4:** Findings on physical and mental health protection

Physical protection	Mental health protection				
Generic measures for Covid‐19 infection prevention^21^, ^22^, ^27^, ^30^, ^32^, ^37^, ^54^, ^55^, ^67^, ^69^, ^76^, ^78^, ^80^	Support from organisations^27^, ^41^, ^42^, ^45^, ^47^, ^52^, ^54^, ^55^, ^62^, ^64^, ^78^, ^79^	Support from team leaders^32^, ^42^, ^47^, ^54^, ^61^	Support from peers^26^, ^30^, ^32^, ^42^, ^62^, ^64^	Support from community^27^, ^53^, ^54^	Self‐support^22^, ^30^, ^32^, ^39^, ^42^, ^61^, ^79^
Approaches to reducing prolonged use of personal protective equipment (PPE)^23^, ^32^, ^36^, ^52^, ^55^, ^60^, ^76^					
Training in physical health protection^17^, ^23^, ^27^, ^32^, ^35^, ^37^, ^38^, ^47^, ^49^, ^53^, ^55^, ^60^, ^61^, ^62^, ^63^, ^66^, ^67^, ^69^, ^73^, ^78^, ^82^					
Strategies for protecting staff^18^, ^22^, ^26^, ^27^, ^30^, ^31^, ^32^, ^37^, ^40^, ^45^, ^53^, ^54^, ^55^, ^59^, ^60^, ^61^, ^66^, ^69^, ^75^, ^78^, ^83^ including those at high risk					

#### 3.4.1 Physical health protection

The findings related to the physical protection of the ICU staff were grouped into the categories that emerged from the analysis. These included generic measures for Covid‐19 infection prevention and control, such as temperature monitoring, strict hand hygiene, personal protective equipment (PPE) use and preservation (e.g.,^32^, ^55^), and vaccination of frontline staff^41^ discussed extensively elsewhere; approaches to reducing prolonged use of PPE (e.g.,^61^, ^68^); training in physical protection (e.g.,^17^, ^36^, ^50^, ^69^) all staff with access to ICUs^61^; as well as strategies for protecting staff, including those at high risk, such as older workers or those with chronic illnesses (e.g.,^45^) (see Table 4).

Considering the discomfort of working in PPE such as overheating, the inability to eat, drink, or use bathroom facilities (e.g.,^51^, ^58^), reducing the extended use of PPE was reported to decrease its potential adverse health effects on ICU HR (e.g.,^68^). These approaches included introducing flexible rostering or shortening the length of the shift to lessen potential fatigue. For example, several ICUs have introduced a four‐shifts‐a‐day system with teams handing over every 6 hours (e.g.,^36^, ^60^, ^63^).

It was recommended to train and frequently re‐train ICU staff in infection control measures (e.g.,^55^) and related topics including inspecting, disinfecting, and safely disposing of PPE to ensure their readiness and effectiveness.^54^ Overall, the reported approaches to training varied and included online trainings, instructional videos,^32^, ^67^ lectures, live demonstrations, or even simulations.^36^, ^66^, ^69^ Several studies also specified that these trainings were developed or organised with the help of professional associations.^27^, ^30^

Various studies discussed strategies for protecting employees (e.g., medical students^40^ or those at high risk^45^, ^83^). These included relocating them to non‐Covid‐19 wards^45^ or providing them (and other ICU staff) with off‐duty observation periods (‘wash‐out’ period) after every period of ward cover if the workforce allowed.^54^, ^82^ Technology including video monitoring, telemedicine, or wearables for vital sign monitoring also emerged as an important tool in allowing staff, including those at high risk, to conduct their regular daily tasks safely and to interact with multidisciplinary teams^18^, ^23^, ^28^, ^30^, ^31^, ^45^, ^53^, ^60^, ^70^, ^75^, ^78^, ^80^ and patients. For example, in some virtual ICUs, physicians, nurses, and specialty consultants (e.g., cardiologists) were able to check the patients via webcam without having to don and doff PPE each time.^28^ Some even proposed to redesign Covid‐19 ICUs, finding that their existing air‐conditioning systems can facilitate the circulation of infected air and suggesting relevant cost‐effective solutions in some specific contexts (e.g., Africa).^83^

#### 3.4.2 Mental health protection

Many studies mentioned that working long shifts with PPE, the infection risk, and frequent ethical decisions regarding care priorities have caused stress and burnout among health professionals (e.g.,^40^, ^41^, ^71^). Nurses, in particular, were reported to be disproportionately affected because they spend a lot of time caring for Covid‐19 patients.^79^

The findings suggest that a number of interventions could be taken to protect the mental well‐being of ICU staff by various actors including organisations, team leaders, colleagues, community, and the health professionals themselves (see Table 4). Thus, organisations can support their ICU staff by providing practical help, including resolving transport and housing issues or setting up psychological counselling or chaplain services^27^, ^41^, ^42^, ^45^, ^47^, ^52^, ^54^, ^55^, ^62^, ^64^, ^78^, ^79^; team leaders by being connected, flexible, human, and present^32^, ^42^, ^47^, ^54^, ^61^; colleagues by providing work‐related support, spot timely signs of concerns such as nightmares or difficulty sleeping, and offer an opportunity to talk^26^, ^30^, ^32^, ^42^, ^62^, ^64^; the community by providing staff with food, cards, or thank you letters sent to care units^27^, ^53^, ^54^; while individuals can support themselves by remembering to eat, drink, sleep, exercise, truthfully report their epidemiological history,^61^ and maintaining regular contact with families and friends.^22^, ^30^, ^32^, ^42^, ^79^

## 4 CONCLUSION

This review is the first known attempt to capture, appraise, and synthesise the corpus of research related to strengthening ICUs' HRM during the Covid‐19 pandemic. The analysis revealed that the existing research is predominantly monodisciplinary, comes primarily from medical journals, and mostly describes the experiences of ICUs within individual health organisations from high‐income countries.

Covid‐19 is claimed to be the greatest challenge for ICUs since these were first established.^69^ Indeed, the findings of this review confirm that it has greatly affected how ICU staff are deployed, managed, and provide care. The emergency highlighted that the healthcare workforce's health and safety must be one of the main priorities of health organisations and spotlighted the role of various actors in supporting health staff in this crisis. However, it was noted during the analysis that the literature examined was mostly silent about broader HRM‐related practices that are also important in the successful realization of organizational change programs in healthcare, such as compensation and rewards, or performance management.^84^ Moreover, and interestingly enough, the literature has not referred explicitly to the important role HRM practitioners play in strengthening the health workforce during Covid‐19 as, for instance, guardians of workplace safety, which has already been declared in the more general literature (e.g.,^85^), and has not represented their perspectives in general. This might be because none of the qualifying papers came from a management‐ or HRM‐oriented journal.

The findings illustrate that to provide care to an unprecedented number of patients who need it, health organisations were often guided by staffing ratios (frequently revisiting them with respect to the Covid‐19 surge), aimed to modify the SOP, redeployed both internal and external staff to ICUs, and created and adapted Covid‐19‐specific staffing models and technological innovations.

Overall, staffing ratios were reported as helpful in matching patients' needs with adequately trained staff and in ensuring patient safety.^74^ This analysis, in line with other relevant research, observed that staffing ratios can vary between contexts (i.e., countries and individual health organisations) and cultures.^1^ It also revealed a number of additional approaches to quantitative workforce planning, while observing that only a few studies focussed to some degree on planning or organising the qualitative aspects of staff.^52^, ^74^ The literature also mostly overlooked the role of HR Information Systems or so‐called People Analytics practices^86^ during the pandemic in helping, for instance, with triangulating data on ‘staff ratios, patient outcomes, and productivity to provide a more evidence‐based approach to workforce planning, and, eventually, learning health systems’.^87^

Prompt relaxation of SOP policies seemed to be crucial in building healthcare workforce capacity during the pandemic.^72^ There were some differences in the literature on whether professionals should work in ICUs outside their regular SOP during the pandemic (unless they had been assessed as skilled). Several qualifying studies reported on this phenomenon (e.g., including the ethical issues related to the providers roles and responsibilities^81^), but none were in‐depth investigations focussing on how the SOP in ICUs had actually been extended or the impact this generated. For example, what was the impact on tasks or job design, or on the adaptation of the healthcare workforce to their extended roles; did it enhance (rather than reduce) health employees' sense of personal or professional identity and the meaningfulness of their roles during Covid‐19? Future studies might like to examine these aspects and provide recommendations on whether and how the roll‐back of SOP amendments should be arranged to ensure organizational justice and mitigate any potential effects on the health providers while acknowledging their sacrifices during the pandemic.^72^

The analysis documented a wide range of physicians, nurses, and other staff who were mobilised to work in ICUs during the pandemic. It was possible to grasp from the literature analysed that the first tranche of redeployed health professionals consisted of volunteers (e.g.,^26^, ^45^). Overall, ethical issues have been raised about decision‐making related to the reallocation of staff to high‐risk clinical roles.^88^ Thus, more studies are also needed to understand the approaches to staff redeployment, and their impact on staff and on patients' health outcomes, and the resources (e.g., other than the training frequently mentioned in qualifying studies) that can help redeployed staff to facilitate the accomplishment of work goals and stimulate personal development and growth. The latter arguments seem to be almost forgotten during the pandemic.

Disasters such as Covid‐19 necessitate and generate innovation. Indeed, the analysis revealed that the pandemic has stimulated the uptake of new approaches to ICU staff planning and care such as virtual care. The former, for example, includes team‐based workforce models or those that consider epidemiological parameters. The uptake of the latter is widely supported by governments and professional organisations, given the potential benefits. For example, in Germany, the law on telemedicine has seen rapid adoption, while in Canada new fee codes for virtual care were fast‐tracked.^1^ The Accreditation Council for Graduate Medical Education in the US has recognized that institutions and training programs are deploying telemedicine to assist trainees in taking care of patients with Covid‐19 remotely,^89^ while the US Food and Drug Administration has cleared a software‐based monitoring platform (Sickbay) that is already used in some ICUs.^28^

Nevertheless, such disasters also raise many questions that can only be answered by the knowledge sharing and collaboration of multidisciplinary teams,^84^ including health management and HRM scholars, to benefit from the knowledge and evidence they accumulated on HRM in crises over the last decades. For example, we need to document and reveal effective change management strategies and leadership types, political and cultural approaches to mobilising staff^1^ and sustaining their motivation and engagement over time as it has become clear that the pandemic is more a marathon than a sprint,^1^ and last but not least to define a ‘new normal’ for the ICUs HR and their HRM. Multi‐disciplinary, multi‐stakeholder, multi‐level, and methodologically pluralist research designs can be adapted^85^ to resolve these dilemmas and effectively promote and facilitate organizational learning, in which employee well‐being is as important as performance. Established HRM theories such as job demands‐resources models can be applied to such studies to investigate how employees devote energy to work activities and/or change activities and their level of work engagement after the launch of organizational changes caused by the Covid‐19 pandemic.

This study has several limitations, as any research does. For example, the limited definition of ICUs may have led to the omission of potentially relevant publications using alternate terminology. The inclusion of materials in English and those published only within the first year of public health emergency may have prevented drawing on relevant studies published in other languages or later periods, respectively. Finally, this research has not considered Covid‐19‐related national plans or reports. Future multi‐lingual scholars might like to analyse these themes, especially from countries where the relevant scholarly literature might be scarce or absent, potentially also broadening the analysis categories used in this study. In addition, future studies could also enrich the findings of this research by studying later corpus of literature.

Despite these limitations, the insights of this research should be helpful for health managers, HRM professionals, and policymakers who face unprecedented challenges and tough decisions during this emergency. There have been various recommendations for HRM‐related actions and interventions in the health sector in response to Covid‐19 (e.g.,^90^), but so far there is not much evidence available on whether and how these recommendations have been enacted. This study revealed state‐of‐the‐art strategies adopted internationally to mobilise ICU staff, revisit SOP, as well as to protect the physical and mental health of employees, which were highlighted as key HRM areas during the pandemic.^16^ It is thus very timely and could inform existing and post‐Covid‐19 ICU HRM policies, help prepare for future health emergencies, and guide and serve as a benchmark for future research.

## CONFLICT OF INTEREST

The authors declare no competing interest.

## Data Availability

Data available on request from the authors.
